# Enhancing Motor Skill Learning with Transcranial Direct Current Stimulation – A Concise Review with Applications to Stroke

**DOI:** 10.3389/fpsyt.2012.00066

**Published:** 2012-07-12

**Authors:** Sangeetha Madhavan, Bhakti Shah

**Affiliations:** ^1^Department of Physical Therapy, University of IllinoisChicago, IL, USA

**Keywords:** tDCS, motor learning, TMS, corticospinal excitability, motor cortex, cortical priming, non-invasive brain stimulation

## Abstract

In the past few years, there has been a rapid increase in the application of non-invasive brain stimulation to study brain-behavior relations in an effort to potentially increase the effectiveness of neuro-rehabilitation. Transcranial direct current stimulation (tDCS), an emerging technique of non-invasive brain stimulation, has shown to produce beneficial neural effects in consequence with improvements in motor behavior. tDCS has gained popularity as it is economical, simple to use, portable, and increases corticospinal excitability without producing any serious side effects. As tDCS has been increasingly investigated as an effective tool for various disorders, numerous improvements, and developments have been proposed with respect to this technique. tDCS has been widely used to identify the functional relevance of particular brain regions in motor skill learning and also to facilitate activity in specific cortical areas involved in motor learning, in turn improving motor function. Understanding the interaction between tDCS and motor learning can lead to important implications for developing various rehabilitation approaches. This paper provides a concise overview of tDCS as a neuromodulatory technique and its interaction with motor learning. The paper further briefly goes through the application of this priming technique in the stroke population.

## Introduction

Non-invasive brain stimulation involves modulation of the central nervous system by electrically activating neurons in the brain (Dymond et al., 1975). The past decade has seen a rapid increase in the application of non-invasive brain stimulation techniques such as transcranial magnetic stimulation (TMS) and transcranial direct current stimulation (tDCS). These neuromodulatory techniques have been widely studied in an effort to provide support for their use as therapeutic adjuvants to enhance functional recovery after impairment. Because of its relative ease of use, portability, and decreased safety risk compared to other neurostimulatory protocols, tDCS is emerging as an effective and versatile clinical tool to prime the neuromotor system prior to or during rehabilitation. As “functional improvement after injury is a relearning process” (Kleim and Jones, 2008), in this review we will provide a brief overview on the application of tDCS to enhance motor skill learning in healthy humans and the physiological mechanisms associated with it. We will also briefly review articles that have used tDCS to enhance motor performance in stroke survivors. Understanding the interaction between tDCS and motor learning can lead to important implications for optimizing neuro-rehabilitation.

## Review Criteria

A search of the literature through January 2012 was performed in the following databases: PubMed, Web of Science, and OVID. The keywords “tDCS and motor learning,” “tDCS and motor performance,” and “tDCS and stroke” were used. Peer reviewed studies were selected if they met the following inclusion criteria: (1) written in English, (2) involved more than one human participant, and (3) included MEP amplitude and/or at least one motor performance-based outcome measure. Because of the vast number of studies that have used tDCS and the presence of numerous review articles on tDCS, we focused on articles relevant to the context of this paper. Data on participants, study design, analysis, follow-up, and outcomes were abstracted. Only studies rated as good or fair by the first author were included. Results were then summarized for the review.

## Parameters of tDCS

Transcranial direct current stimulation involves delivering a low intensity direct current between two sponge electrodes, which are typically moistened with NaCl solution and placed on the scalp (Priori et al., 1998; Nitsche and Paulus, 2000). NaCl solution is usually preferred as it minimizes discomfort (Dundas et al., 2007). The active electrode (size between 5 and 35 cm^2^) is conventionally placed on the area of the brain to be stimulated. The other electrode (usually equal or larger size than the active electrode) is placed on a region contralateral to this placement; such as the forehead if the stimulated area is the primary motor cortex. Typically current intensities of 0.5–2 mA are applied for a duration of 5–20 min, yielding a current density of 0.02–1 mA/cm^2^, and a total charge between 15 and 100 μC/cm^2^.

The efficacy of tDCS depends on current density which determines the induced electrical field strength (Parazzini et al., 2011). Depending on electrode dimensions, position, and the current density, approximately half of the current injected during tDCS is shunted through the scalp (Miranda et al., 2006; Sadleir et al., 2010). Low intensity currents when applied for even short periods of 10–13 min have shown to induce cortical excitability changes lasting up to 90 min (Nitsche and Paulus, 2000, 2001). These changes in corticospinal excitability, as measured by TMS evoked motor potentials, range from 40 to 150% above baseline values for anodal stimulation, and 20 to 50% below baseline values for cathodal stimulation (Nitsche and Paulus, 2000, 2001). In order to induce after effects, Nitsche and Paulus (2000) showed that a stimulus duration of at least 3 min at 1 mA or an intensity of 0.6 mA for 5 min is required. Anodal stimulation typical enhances cortical excitability while cathodal stimulation decreases excitability (Nitsche and Paulus, 2001; Nitsche et al., 2003b). These effects have been more robust in the upper limb representations compared to the lower limb motor representations.

## Safety and Adverse Effects of tDCS

Because tDCS does not involve direct brain-electrode interface and uses low currents, it can be used safely without adverse risks. The density and charge values that are conventionally used compare well with two safety studies that reported no neural damage or change in cognitive function with applied current charges less than 96 μC/cm^2^ (Nitsche and Paulus, 2001; Iyer et al., 2005). However special care should be taken if the patient has alteration of the skull, such as trepanation or fracture, or if the patient has decreased integrity to the skin surface, as it may result in tissue damage because of increased current densities.

During current application, neurally driven vasodilatation may result in transient mild redness below the electrode surface (Durand et al., 2002). A study by Poreisz et al. (2007), focusing on the safety aspects of tDCS, reported a mild tingling sensation as the most common adverse effect; observed by 71% of the subjects during and 8% after the stimulation. Moderate fatigue was the second frequent adverse effect and a light itching sensation under the electrodes occurred in 30% of the subjects during the stimulation and in 15% after the stimulation. Very few subjects felt a slight burning sensation or a mild pain sensation under the electrodes. About 11% reported difficulties in concentrating during tDCS whereas headache seemed to occur in 5% of cases during and 12% after stimulation.

Care should be taken that there are no metallic implants near the electrodes. None of the tDCS studies have reported any serious complications of stimulation, such as seizure or instance of psychotic symptoms. However, it is necessary to keep in mind that anodal tDCS increases cortical excitability and it may be prudent to exclude patients at risk for epileptic seizures. In addition, repetitive application and long durations of tDCS should be carefully monitored for adverse effects even though none have been reported so far. Thus, to the best of our knowledge, tDCS protocols that have been conventionally used appear to be safe and the side effects are commonly limited to focal tingling, itching, and a local erythema, making it a preferred technique of choice.

## Mechanism of Action

### During stimulation

During stimulation, effects of tDCS are primarily based on the principle of modulation of neuronal membrane potential, altering the conductance of sodium and calcium channels. Depending on the polarity of stimulation (anodal vs. cathodal), tDCS induces spontaneous neuronal excitability by a tonic depolarization or hyperpolarization of the resting membrane potential (Creutzfeldt et al., 1962; Purpura and McMurtry, 1965). Anodal (positive) stimulation increases the spontaneous firing rate and the excitability of cortical neurons by depolarizing the membranes, while cathodal (negative) stimulation leads to hyperpolarization of the neuronal membranes resulting in decreased neuronal firing rate and excitability. In addition to polarity changes in the superficial membranes, Creutzfeldt et al. (1962) demonstrated that neurons in the deeper layers of the cat motor cortex are stimulated by cathodal and inhibited by anodal stimulation, probably as a result of the inversion of current flow associated with the neuron’s spatial orientation. Hence it is important to keep in mind that tDCS could create dissimilar levels of polarity in the deeper layers.

### Post-stimulation

The after effects of tDCS are not simply because of prolonged membrane potential shifts, but also due to mechanisms similar to long-term potentiation (LTP) and long-term depression (LTD) (Islam et al., 1995; Nitsche and Paulus, 2000; Nitsche et al., 2003a). NMDA-receptor modulation is involved in the induction of LTP- and LTD-like mechanisms. Activation of the NMDA receptors results in an increase in intracellular calcium in the post synaptic neuron. A small increase in the post synaptic calcium levels leads to LTD- and a greater increase induces LTP-like mechanisms (Lisman, 2001). After effects of tDCS are presumably driven by the activation of the NMDA receptors. Dextromethorphan (DMO), a NMDA-receptor antagonist has been reported to suppress the post-stimulation effects of both anodal and cathodal stimulation (Liebetanz et al., 2002; Nitsche et al., 2003a). NMDA-receptor efficacy depends on intracellular calcium level (a prolonged calcium increase enhances NMDA-receptor efficacy, while a low calcium level reduces it; Bennett, 2000; Lisman, 2001). By applying calcium channel blockers during stimulation, Nitsche et al. (2003a) showed that tDCS elicits modifications in NMDA receptors via changes in intracellular calcium concentration.

Evidence also suggests the involvement of inhibitory GABAergic synapses for the after effects of tDCS. Using a paired pulse TMS protocol to measure intracortical inhibition, Nitsche et al. (2005) reported a prominent involvement of intracortical inhibitory mechanisms for the resulting excitability modulations. Anodal tDCS resulted in a reduction of short latency intracortical inhibition and an increase in indirect wave (I-wave) facilitation, suggesting a decrease in the GABA interneuronal activity. Stagg et al. (2009) further demonstrated that 10 min of anodal tDCS significantly decreases GABA concentration. The after effects of cathodal tDCS are also dependent on modulation of GABAergic and in addition glutamatergic synapses. In the above mentioned paired pulse TMS study by Nitsche et al. (2005), cathodal stimulation led to a significant decrease in intracortical facilitation. Stagg et al. (2009) reported that the concentration of glutamate was significantly decreased within the cathodally stimulated cortex. In addition to NMDA, GABA, and glutamate involvement, the after effects of tDCS are also modulated by serotonin, dopamine, and acetylcholine (Kuo et al., 2007, 2008; Nitsche et al., 2009a,b). More recently, tDCS has also been reported to enhance brain derived neurotrophic factor (BDNF) secretion and tyrosine receptor kinase B (TrkB) activation which are also critical factors for augmentation of synaptic plasticity and motor learning (Fritsch et al., 2010).

Although the mechanisms of action of tDCS are not yet completely understood, we can conclude that tDCS not only alters spontaneous neuronal firing rate by altering the resting membrane potential, but it helps to produce neuroplastic changes by altering synaptic function.

## Transcranial Direct Current Stimulation and Motor Skill Learning in Individuals without Neurological Disorders

Motor skill learning refers to the process by which movements are executed more quickly, accurately, and efficiently with practice (Willingham, 1998). Technological and methodological advances in neuroimaging as well as non-invasive brain stimulation have provided us with a greater understanding of the neural substrates involved in skill acquisition. Motor skill learning is typically characterized by increased functional connectivity in a distributed network that involves the primary motor (M1), premotor, and supplementary motor cortices, the cerebellum, thalamic nuclei, and the striatum (Honda et al., 1998; Ungerleider et al., 2002; Seidler, 2010). At the neural level, motor skill learning is accompanied by changes in neuronal activity and excitability, and synaptic plasticity (Dayan and Cohen, 2011). Mechanisms like LTP and LTD are widely considered major cellular mechanisms underlying learning and memory (Bliss and Collingridge, 1993; Rioult-Pedotti et al., 2000).

The physiological basis of tDCS, as described earlier, is analogous to the mechanisms that accompany motor learning. As the M1 is involved critically in motor skill learning (Shmuelof and Krakauer, 2011), many studies have targeted the M1 to facilitate motor learning processes using tDCS, either by enhancing excitability in the learning M1 using anodal stimulation or by decreasing excitability in the resting M1 via cathodal stimulation. In the following section we present animal and human studies that have applied tDCS to the healthy brain to augment motor skill learning.

### tDCS of the upper limb motor cortex

Early studies on primates have associated anodal tDCS of the cortical surface with improved learning (Rosen and Stamm, 1972). Almost two decades later, Nitsche et al. (2003c) studied the effects of tDCS on implicit motor learning in humans by exploring hand motor performance during a variant of the serial reaction time task. tDCS was applied separately to the M1, premotor, and prefrontal cortices during performance of the motor task. It was found that the reaction time of the skilled task decreased during facilitatory anodal tDCS stimulation compared to inhibitory cathodal or sham stimulation. This improvement was primarily noted during stimulation of the M1 and not the other areas. These results have also been supported by many other studies, which have shown that increasing excitability of the learning M1 using anodal stimulation leads to improvements in motor learning (Jaeger et al., 1987; Boggio et al., 2006). More recently, Stagg et al. (2011) examined the effects of tDCS over the M1 during an explicit motor learning task consisting of sequential finger presses. Similar to previous motor learning studies on implicit behavior, they showed that application of tDCS during motor practice led to modulation of behavior in a polarity specific manner as compared to sham in which anodal tDCS led to faster learning and cathodal tDCS slowed down learning. In this study tDCS was found to modulate both the total amount of learning as well as the rate of learning.

Reis et al. (2009) examined repeated applications of anodal tDCS on M1 during motor skill learning over five consecutive days. A skill measure that reflected shifts in the task’s speed–accuracy tradeoff was chosen. Anodal tDCS not only led to significant greater total learning but the enhanced skill measure remained superior in the anodal group compared to sham tDCS even at 3 months, suggesting that tDCS not only enhances motor learning but can also positively influences long-term consolidation. In addition to enhancing motor learning, de Xivry et al. (2011) demonstrated that anodal tDCS of the M1 also has the capacity to enhance generalization of learning. In this study, healthy participants adapted to a force field by reaching to a single target in one trained direction and were later tested for generalization in another workspace. Interestingly stimulation of the M1 (and not the adjacent posterior parietal cortex) enhanced the generalization process in the intrinsic coordinates of the joints and muscles but did not affect the extrinsic coordinates (environment), a finding highly relevant to rehabilitation.

Anodal tDCS over the M1 during motor practice has also been shown to enhance coding and retention of motor memory (Galea and Celnik, 2009). However, when anodal TDCS is applied during the last phase of motor training, it is shown to have a negative effect on motor memory formation (Rosenkranz et al., 2000). Similar studies using repetitive TMS have shown that the same TMS protocol can be facilitatory or inhibitory depending on the prior state of the system (Siebner et al., 2004; Iezzi et al., 2008; Kantak et al., 2010). Hence it is possible that the effects of tDCS also depend on the prior state of the corticomotor system.

Fritsch et al. (2010) examined molecular mechanisms underlying the effect of tDCS on motor skill learning using a mouse model. This group showed that tDCS is beneficial to motor learning when BDNF release occurs through training and that in the absence of activity-dependent BDNF secretion (conditional BDNF knock-out mice), the beneficial effects of tDCS may not materialize. They further reported that anodal tDCS with combined repetitive low-frequency synaptic activation induces LTP that is NMDA-receptor dependent and mediated by secretion of BDNF.

### tDCS of the visual cortex

In addition to its effects on the M1, tDCS has also shown to have effects on the extrastriate visual area (V5) known to mediate motion processing and contribute to visuo-motor learning. Antal et al. (2004) tested visuo-motor learning by enhancing the excitability of the M1, the primary visual cortex, and the extra striate visual area (V5) using anodal tDCS with three different electrode configurations in different groups of subjects. Facilitatory stimulation of the M1 and V5 resulted in improved performance during the early learning phase of the visually guided manual tracking task. Cathodal stimulation did not show any effect. As visuo-motor tasks highly depend on visual perception and cognitive processing, tDCS could possibly modulate either of these processes contributing to motor learning.

### tDCS of the dorsolateral prefrontal cortex

Memory enhancement is of interest to those involved in rehabilitation as behavioral changes during learning are implemented by memory processes in the brain (Maxwell et al., 2003; Kantak and Winstein, 2012). There is evidence that anodal tDCS of the dorsolateral prefrontal cortex (DLPFC) enhances working memory. Working memory refers to temporary storage of information to be made available for future information processing. Working memory is also crucial to many higher-order strategic functions and plays a central role in long-term memory. The DLPFC plays a crucial role during working memory tasks (Butefisch et al., 2004). Fregni et al. (2005) investigated the effects of anodal stimulation of the DLPFC on working memory. Subjects performed a three-back working memory tasks based on letters during tDCS application over the DLPFC. Although there was no significant difference with respect to the response time, results showed increased correct responses and less errors with anodal stimulation of DLPFC compared to cathodal or sham of the DLPFC, or anodal stimulation of the M1. Zaehle et al. (2011) added further evidence to the interaction between tDCS and working memory by investigating the modulatory effects of tDCS on the underlying oscillatory brain activity with electroencephalography (EEG). At the level of neural ensemble, synchronized activity of a large number of neurons gives rise to oscillations that can be observed using EEG. Using a two-back letter memory task, the authors found a significant effect of stimulation in which the participants responded faster and showed improved performance after anodal tDCS compared to sham and cathodal tDCS. An increase in oscillatory power after anodal tDCS and a decrease in oscillatory power after cathodal tDCS was observed. Changes in oscillatory brain activity play an important role in the formation of perception and memory and thus are essential for higher cognitive functions. This study highlights the potential application of tDCS in pathologies that have not only been associated with memory deficits but also involve alterations of oscillatory brain activity.

### tDCS of the cerebellum

A few recent studies have targeted the cerebellum for the application of tDCS as it is a critical structure involved in movement control and cognitive processing. Galea et al. (2009) showed that tDCS is capable of modifying cerebellar excitability. Cathodal tDCS decreased and anodal tDCS increased cerebellar inhibition of M1. This change in excitability lasted for 30 min after stimulation and did not affect the excitability of the brainstem or corticomotor system. Anodal cerebellar tDCS also helped subjects adapt faster to a novel visuo-motor transformation paradigm compared to M1 stimulation. However, tDCS of the M1 resulted in a marked increase in retention of the task (Galea et al., 2011).

### tDCS of lower limb areas

All of the studies mentioned above are related to upper limb motor tasks. Studies examining the effects of tDCS on lower limb motor learning are limited. Because of the proximity of the two lower limb motor cortices, targeting tDCS to one hemisphere without inducing a same sign modulation in the opposite hemisphere is a challenge. Using a combination of carefully selected electrode size and position, Madhavan and Stinear (2010) successfully applied tDCS to one lower limb M1 while creating an opposite sign modulation in the other M1. They also noted that it was possible to focally up regulate one hemisphere in almost 80% of subjects tested. Although anodal tDCS has shown to successfully enhance cortical excitability of the lower limb muscle representations, cathodal tDCS does not reveal the expected downregulation of excitability that is commonly reported in upper limb studies (Jeffery et al., 2007).

Tanaka et al. (2009) applied anodal tDCS to the lower limb M1 and showed that the facilitatory tDCS can improve maximal leg pinch force and that this improvement is retained for approximately 30 min after the end of stimulation. This effect was specific only to leg motor performance and did not influence hand function suggesting spatial specificity of the effects of tDCS. Recently, Jayaram et al. (2012) showed that cerebellar tDCS can increase or decrease the rate of adaptation to a novel task depending on anodal or cathodal tDCS (respectively) over the cerebellum during a specific cerebellar-dependant locomotor training paradigm.

To summarize, tDCS applications in the healthy brain open up the possibilities of using tDCS as an experimental and rehabilitation tool for understanding and improving upper extremity and lower extremity motor function and learning.

## Use of tDCS for Functional Recovery in Stroke

Relearning of motor skills is a fundamental process for recovering motor function after neurological injury such as stroke (Carr and Shepherd, 1987; Kleim and Jones, 2008). Learning is required for both recovery (restoring the ability to perform movement in the same manner as it was performed prior to injury) and compensation (performing a task in a manner different from how it was performed prior to injury (Krakauer, 2006; Kleim, 2011)). Since it is still debated whether individuals with stroke have true motor learning deficits, and whether recovery from stroke is indeed a form of model-free motor learning (Krakauer, 2006), in this paper we choose to focus on the application of tDCS in stroke in the context of training-induced improvements in motor function as most papers cited below have tested only changes in motor function in stroke patients after single or repeated applications of tDCS.

The use of tDCS in stroke is based on the model of inter-hemispheric imbalance. In healthy individuals, balance between-hemisphere corticospinal excitability is maintained via transcallosal inhibitory connections, whereby each hemisphere acts to inhibit the other. Typically after stroke, the non-lesioned M1 becomes hyperexcitable because of decreased transcallosal inhibition imposed by the lesioned hemisphere (Traversa et al., 1998). Primarily this imbalance in between-hemisphere corticospinal excitability is suggested to be maladaptive and a marker of poor functional recovery (Rossini et al., 2003; Ward et al., 2003; Serrien et al., 2004; Duque et al., 2007; Madhavan and Stinear, 2010). Further evidence for this can be found in training studies where post-training improvement in upper limb and lower limb motor function is associated with a decrease in the excitability of the non-lesioned M1 or increase in the excitability of the lesioned M1 (Muellbacher et al., 2002; Lin et al., 2008; Yen et al., 2008). Consistent with this idea, many have used tDCS to manipulate cortical excitability to facilitate motor learning and enhance the effects of traditional therapy. Up regulating the lesioned hemisphere with excitatory stimulation or down regulating the non-lesioned M1 with inhibitory stimulation may help redress the symmetry in between-hemisphere corticomotor excitability and enhance motor learning. Cortical stimulation, in combination with a suitable motor therapy, may be a new treatment option to increase the effectiveness of rehabilitation (Krakauer et al., 2012). The idea behind this approach is that combined peripheral activities and central brain stimulation can enhance synaptic plasticity and motor skill acquisition by modulating the afferent inputs to the cortex when it is centrally stimulated.

### Upper limb

Numerous studies have reported the beneficial effects of anodal tDCS on the lesioned M1 or cathodal tDCS over the non-lesioned M1 in improving motor performance of the affected limb in patients after stroke. Details of these studies are characterized in Table 1. Ten to fifteen minutes of tDCS before or during performance of skilled movement tasks has resulted in approximately 10–20% improvement in paretic upper limb motor function after single or multiple sessions of tDCS. In some studies (particularly those with repeated stimulation), the effects have outlasted stimulation from 24 h up to 6 months (Boggio et al., 2007; Hesse et al., 2007; Kim et al., 2010; Bolognini et al., 2011; Nair et al., 2011; Zimerman et al., 2012). Most of these studies have used upper limb motor performance measures such as Jebsen Taylor Hand Function Test and Upper Extremity Fugl-Meyer Scale. Although these studies provide valuable data regarding changes in clinical function, not much information on the neural or motor mechanisms that resulted in these improvements is reported. It should also be noted that the magnitude of improvement is varied among studies and may be dependent on the dosage of tDCS delivered, the type of patients recruited (acute, sub-acute, or chronic), outcome measure used and the type of task performed in conjunction with tDCS. As in many stroke studies, not all subjects have shown the expected improvement.

**Table 1:** **Summary of study characteristics**.

Studies included	Sample population	Study design	Type of tDCS	Number of sessions	Time of testing	Outcome measure	Change in performance
Zimerman et al. (2012)	Chronic Stroke	Double blind, crossover, sham controlled	Cathodal during training	Single	During, 90 min and 24 h after the intervention	Performance of a complex sequential finger movement task of paretic hand	Improvement
Rossi et al. (2012)	Acute Stroke	Double blind, sham controlled	Anodal during rest	Repeated (5 daily sessions)	Onset, at 5 days and after 3 months	(1) Short form of Fugl-Meyer motor scale; (2) National Institute of Health Stroke Scale; (3) Barthel Index; (4) Modified Rankin Scale	No difference
Nair et al. (2011)	Chronic stroke	Double blind, sham controlled	Cathodal combined with OT for paretic arm and hand	Repeated (5 daily sessions)	(1) ROM: baseline, after 5 days and again 7 days later; (2) upper Extremity – Fugl-Meyer: baseline and 7 days after intervention	(1) ROM for upper extremity; (2) Upper Extremity Fugl-Meyer	Improvement
Bolognini et al. (2011)	Chronic Stroke	Double blind, sham controlled	Bi-hemispheric followed by CIMT for paretic hand	Repeated (10 daily sessions)	Baseline, day 1, day 5, day 10 (end of treatment) and at 2 and 4 weeks follow-up	(1) Jebson Taylor Test; (2) Hand grip strength; (3) Motor Activity Log Scale; (4) Fugl-Meyer Motor scale	Improvement
Tanaka et al. (2011)	Chronic Stroke	Double blind, crossover, sham controlled	Anodal during paretic quadriceps contraction	Single	Before, during, and 30 min after tDCS	Maximal knee-extension force measured with handheld dynamometer	Improvement
Madhavan et al. (2011)	Chronic Stroke	Single blind, crossover, sham controlled	Anodal during paretic ankle target tracking	Single	Before, during, and after	Tracking accuracy	Improvement
Geroin et al. (2011)	Chronic Stroke	Pilot randomized clinical trial	Anodal combined with robot assisted gait training	Repeated (10 sessions over 2 weeks)	Baseline, immediately after and 2 weeks post treatment	(1) Six-minute walking test (2) 10-m walking test	No difference
Lindenberg et al. (2010)	Chronic Stroke	Sham controlled	Bi-hemispheric combined with PT/OT of paretic upper limb	Repeated (5 daily sessions)	Baseline, after 3 and 7 days post intervention	(1) Upper Extremity Fugl-Meyer; (2) Wolf Motor Function Test	Improvement
Kim et al. (2010)	Sub-acute Stroke	Single blinded, sham controlled	Anodal, cathodal, or sham combined with OT of paretic upper limb	Repeated (10 days sessions)	Baseline, 1 day after and 6 months after	(1) Upper Extremity Fugl-Meyer; (2) Barthel Index	Improvement
Boggio et al. (2007)	Chronic Stroke	Exp 1: double blind, cross over, sham controlled,	Exp 1: anodal, cathodal, and sham at rest Exp 2	Exp 1: repeated (4 weekly sessions) Exp 2: 5	Exp 1: baseline, following session 1 and session 4. Exp 2: baseline	Jebson Taylor Test	Improvement
		Exp 2: open label study	Cathodal at rest	Consecutive daily sessions	During and 1 and 2weeks after intervention		
Hesse et al. (2007)	Sub-acute Stroke	Pre–post	Anodal tDCS with robot assisted arm training of paretic limb	Repeated (30 sessions over 6 weeks)	Pre–post	(1) Upper extremity Fugl-Meyer; (2) Aachener Aphasia Test	Mixed results (only 3/10 improved)
Hummel et al. (2005)	Chronic Stroke	Double blind, crossover, sham controlled	Anodal during Jebsen Taylor test	Single	Baseline, during, post and 10 days after intervention	Jebson Taylor Test	Improvement

*ROM, range of motion; OT, occupational therapy; PT, physical therapy; CIMT, constrained induced movement therapy; tDCS, transcranial direct current stimulation*.

### Lower limb

There is relatively less evidence for the effects of tDCS on lower limb motor function post stroke. Madhavan et al. (2011) were the first to report purposeful modulation of ankle motor practice in stroke patients after facilitatory stimulation of the lesioned lower limb M1. This improvement with motor practice seen with anodal stimulation was not observed with sham stimulation or anodal stimulation of the non-lesioned lower limb M1, emphasizing the polarity specific and focal effects of tDCS. Similarly, Tanaka et al. (2011) showed that a single session of facilitatory tDCS is capable of enhancing quadriceps extensor force in chronic patients. Despite the promising preliminary effects of tDCS on lower limb motor control and strength, in contrast Geroin et al. (2011) found that applying anodal tDCS in combination with robotic gait training did not enhance the effects of robotic gait training in stroke patients. Whether a different dosage of stimulation or combining tDCS with a different gait training paradigm will be beneficial to enhance outcomes of gait training is yet to be determined.

### Bi-hemispheric stimulation

Studies are also now beginning to examine the effects of bi-hemispheric brain stimulation using tDCS. Lindenberg et al. (2010) investigated whether tDCS modulation of bilateral motor cortices in combination with physical and occupational therapy improves motor outcome after stroke. They used anodal tDCS to upregulate excitability of lesioned M1 and cathodal tDCS to downregulate excitability of the non-lesioned M1. A significant improvement (~20%) in motor function scores was seen with simultaneous bilateral modulation. The effects outlasted the stimulation by at least 1 week. The authors suggested that cathodal stimulation helps augment the direct effects of the anodal stimulation through additional modulation of inter-hemispheric interactions.

In summary, tDCS has revealed preliminary success in enhancing motor learning and recovery in stroke patients. tDCS has a greater advantage over other non-invasive brain stimulation techniques in a clinical setting because of its low-cost (approximately $500 per device), ease of use, portability, and low risk. However, individualized options with tDCS need to be investigated in patients especially regarding dosage of current, site of stimulation, time of window of stimulation, and type of therapy to perform in conjunction with stimulation. It is also important to consider the residual anatomical and physiological substrates available to each patient before prescribing tDCS. Upregulating the lesioned M1 or down regulating the non-lesioned M1 may not necessarily be the optimal approach for all patients. For example: if a patient’s anatomical resources in the lesioned hemisphere are limited, then suppressing the non-lesioned M1 may be of concern and upregulation of the non-lesioned M1 could be an option. This is a hypothesis that needs to be tested.

## Limitations

Although most of the studies presented above depict an optimal picture of desired modulation of cortical excitability in conjunction with improvements in motor performance and motor learning, it is necessary to remember that tDCS research is still in its preliminary stage and has several associated caveats: (1) Most previous investigations have focused on short-term improvements in performance and learning. Larger experimental and clinical trials are required to assess the effects of repeated applications of tDCS in association with multiple training sessions, their interaction with specific motor learning stages and tasks, and the extent to which these performance improvements cause clinical changes and aspects of safety. (2) For the successful implementation of this technique as an interventional strategy, a better understanding of the underlying neurophysiological mechanisms is essential. (3) Because of inter-individual differences in conductivity, scalp resistance, and orientation of the cortical neurons, precise current flow cannot be predicted. Indeed, there is substantial variability in the after effects of tDCS. Madhavan et al. (2010) found that the same dosage of facilitatory stimulation that induced upregulation in majority of subjects downregulated cortical excitability of the lower limb motor cortex for some. Hence, some measure of cortical excitability is needed to ensure that the desired upregulation was obtained. (4) There is a need for more research regarding electrode and current parameters to hone the temporal and spatial resolution of tDCS. (5) It is yet to be clear whether the effects of tDCS are optimal during online (during task performance) vs. offline (before or after task performance) stimulation. Stagg et al. (2011) showed that the application of anodal tDCS before a sequence-learning task resulted in slower learning. The importance of such timing dependence has not yet been fully explored for tDCS. (6) Most of the studies of tDCS in stroke patients have been limited to sub-acute and chronic stages of recovery. A recent study by Rossi et al. (2012) found that repeated sessions of anodal tDCS to the lesioned motor cortex applied during rest in acute stroke patients did not accelerate function recovery. Whether this was a function of application during rest instead of motor practice or the responsivity of the time of stroke is yet to be determined.

## Conclusion and Future Directions

Despite the above limitations, the following can be concluded about tDCS:

- There is accumulating evidence to suggest that tDCS is effective in modulating cortical excitability in most cases. Typically, anodal tDCS increases neuronal excitability and cathodal tDCS decreases neuronal excitability. As the desired modulation may not be obtained in every individual, electrophysiological measures should be included to establish the desired sign and extent of modulation especially when using this as an adjuvant to therapy.
- Upregulation of the lesioned hemisphere and/or downregulation of the non-lesioned hemisphere appears to enhance the outcomes of rehabilitation in stroke patients. tDCS is typically applied before or in conjunction with a motor task to optimize training outcomes. Hence, it is important to consider it as an adjuvant to prime the brain and not therapy itself.
- The area of stimulation should be chosen depending on the expected outcome. For e.g., facilitatory stimulation of the M1 may help better retention of a skilled motor task while stimulation over the cerebellum may help with faster adaptation to the task (Galea et al., 2009).
- To the best of our knowledge, tDCS used within conventional parameters appears to be low risk and can be used without adverse effects in patients.

In conclusion, tDCS offers a low-cost, portable, and potentially high-impact option for enhancing skilled motor learning and neuro-rehabilitation. Larger randomized controlled trials are needed for the design and optimization of tDCS as a therapeutic tool for patients after stroke.

## Conflict of Interest Statement

The authors declare that the research was conducted in the absence of any commercial or financial relationships that could be construed as a potential conflict of interest.

## References

[B1] Antal A. Nitsche M. A. Kincses T. Z. Kruse W. Hoffmann K. P. Paulus W. (2004). Facilitation of visuo-motor learning by transcranial direct current stimulation of the motor and extrastriate visual areas in humans. Eur. J. Neurosci. 19, 2888–2892 10.1111/j.1460-9568.2004.03367.x 15147322

[B2] Bennett M. R. (2000). The concept of long term potentiation of transmission at synapses. Prog. Neurobiol. 60, 109–137 10.1016/S0301-0082(99)00040-4 10639051

[B3] Bliss T. V. P. Collingridge G. L. (1993). A synaptic model of memory – long-term potentiation in the hippocampus. Nature 361, 31–39 10.1038/361031a0 8421494

[B4] Boggio P. S. Castro L. O. Savagim E. A. Braite R. Cruz V. C. Rocha R. R. Rigonatti S. P. Silva M. T. Fregni F. (2006). Enhancement of non-dominant hand motor function by anodal transcranial direct current stimulation. Neurosci. Lett. 404, 232–236 10.1016/j.neulet.2006.05.051 16808997

[B5] Boggio P. S. Nunes A. Rigonatti S. P. Nitsche M. A. Pascual-Leone A. Fregni F. (2007). Repeated sessions of noninvasive brain DC stimulation is associated with motor function improvement in stroke patients. Restor. Neurol. Neurosci. 25, 123–129 17726271

[B6] Bolognini N. Vallar G. Casati C. Latif L. A. El-Nazer R. Williams J. Banco E. Macea D. D. Tesio L. Chessa C. Fregni F. (2011). Neurophysiological and behavioral effects of tDCS combined with constraint-induced movement therapy in poststroke patients. Neurorehabil. Neural Repair 25, 819–829 10.1177/1545968311411056 21803933

[B7] Butefisch C. M. Khurana V. Kopylev L. Cohen L. G. (2004). Enhancing encoding of a motor memory in the primary motor cortex by cortical stimulation. J. Neurophysiol. 91, 2110–2116 10.1152/jn.01038.2003 14711974

[B8] Carr J. Shepherd R. (1987). “A motor learning model for rehabilitation,” in Movement Science: Foundations for Physical Therapy in Rehabilitation, eds Carr J. H. Shepherd R. B. Gordon J. Gentille A. M. Held J. N. (Rockville, MD: Aspen), 31–91

[B9] Creutzfeldt O. D. Fromm G. H. Kapp H. (1962). Influence of transcortical d-c currents on cortical neuronal activity. Exp. Neurol. 5, 436–452 10.1016/0014-4886(62)90056-0 13882165

[B10] Dayan E. Cohen L. G. (2011). Neuroplasticity subserving motor skill learning. Neuron 72, 443–454 10.1016/j.neuron.2011.10.008 22078504 PMC3217208

[B11] de Xivry J. J. Marko M. K. Pekny S. E. Pastor D. Izawa J. Celnik P. Shadmehr R. (2011). Stimulation of the human motor cortex alters generalization patterns of motor learning. J. Neurosci. 31, 7102–7110 10.1523/JNEUROSCI.0273-11.2011 21562272 PMC3330486

[B12] Dundas J. E. Thickbroom G. W. Mastaglia F. L. (2007). Perception of comfort during transcranial DC stimulation: effect of NaCl solution concentration applied to sponge electrodes. Clin. Neurophysiol. 118, 1166–1170 10.1016/j.clinph.2007.01.010 17329167

[B13] Duque J. Murase N. Celnik P. Hummel F. Harris-Love M. Mazzocchio R. Olivier E. Cohen L. G. (2007). Intermanual differences in movement-related interhemispheric inhibition. J. Cogn. Neurosci. 19, 204–213 10.1162/jocn.2007.19.2.204 17280510

[B14] Durand S. Fromy B. Bouye P. Saumet J. L. Abraham P. (2002). Vasodilatation in response to repeated anodal current application in the human skin relies on aspirin-sensitive mechanisms. J. Physiol. (Lond.) 540, 261–269 10.1113/jphysiol.2001.013364 11927685 PMC2290218

[B15] Dymond A. Coger R. Serafetinides E. (1975). Intracerebral current levels in man during electrosleep therapy. Biol. Psychiatry 10, 101–104 1120172

[B16] Fregni F. Boggio P. S. Nitsche M. Bermpohl F. Antal A. Feredoes E. Marcolin M. A. Rigonatti S. P. Silva M. T. A. Paulus W. Pascual-Leone A. (2005). Anodal transcranial direct current stimulation of prefrontal cortex enhances working memory. Exp. Brain Res. 166, 23–30 10.1007/s00221-005-2334-6 15999258

[B17] Fritsch B. Reis J. Martinowich K. Schambra H. M. Ji Y. Cohen L. G. Lu B. (2010). Direct current stimulation promotes BDNF-dependent synaptic plasticity: potential implications for motor learning. Neuron 66, 198–204 10.1016/j.neuron.2010.03.035 20434997 PMC2864780

[B18] Galea J. M. Celnik P. (2009). Brain polarization enhances the formation and retention of motor memories. J. Neurophysiol. 102, 294–301 10.1152/jn.00184.2009 19386757 PMC2712265

[B19] Galea J. M. Jayaram G. Ajagbe L. Celnik P. (2009). Modulation of cerebellar excitability by polarity-specific noninvasive direct current stimulation. J. Neurosci. 29, 9115–9122 10.1523/JNEUROSCI.2184-09.2009 19605648 PMC2760225

[B20] Galea J. M. Vazquez A. Pasricha N. Orban De Xivry J. J. Celnik P. (2011). Dissociating the roles of the cerebellum and motor cortex during adaptive learning: the motor cortex retains what the cerebellum learns. Cereb. Cortex 21, 1761–1770 10.1093/cercor/bhq246 21139077 PMC3138512

[B21] Geroin C. Picelli A. Munari D. Waldner A. Tomelleri C. Smania N. (2011). Combined transcranial direct current stimulation and robot-assisted gait training in patients with chronic stroke: a preliminary comparison. Clin. Rehabil. 25, 537–548 10.1177/0269215510389497 21402651

[B22] Hesse S. Werner C. Schonhardt E. M. Bardeleben A. Jenrich W. Kirker S. G. (2007). Combined transcranial direct current stimulation and robot-assisted arm training in subacute stroke patients: a pilot study. Restor. Neurol. Neurosci. 25, 9–15 17473391

[B23] Honda M. Deiber M. P. Ibanez V. Pascual-Leone A. Zhuang P. Hallett M. (1998). Dynamic cortical involvement in implicit and explicit motor sequence learning. A PET study. Brain 121(Pt 11), 2159–2173 10.1093/brain/121.11.2159 9827775

[B24] Hummel F. Celnik P. Giraux P. Floel A. Wu W. H. Gerloff C. Cohen L. G. (2005). Effects of non-invasive cortical stimulation on skilled motor function in chronic stroke. Brain 128, 490–499 10.1093/brain/awh369 15634731

[B25] Iezzi E. Conte A. Suppa A. Agostino R. Dinapoli L. Scontrini A. Berardelli A. (2008). Phasic voluntary movements reverse the after effects of subsequent theta-burst stimulation in humans. J. Neurophysiol. 100, 2070–2076 10.1152/jn.90521.2008 18753328

[B26] Islam N. Aftabuddin M. Moriwaki A. Hattori Y. Hori Y. (1995). Increase in the calcium level following anodal polarization in the rat-brain. Brain Res. 684, 206–208 10.1016/0006-8993(95)00434-R 7583224

[B27] Iyer M. B. Mattu U. Grafman J. Lomarev M. Sato S. Wassermann E. M. (2005). Safety and cognitive effect of frontal DC brain polarization in healthy individuals. Neurology 64, 872–875 10.1212/01.WNL.0000152986.07469.E9 15753425

[B28] Jaeger D. Elbert T. Lutzenberger W. Birbauner N. (1987). The effects of externally applied transcephalic direct currents on lateralization in choice reaction tasks. J. Psychophsiol. 127–133

[B29] Jayaram G. Tang B. Pallegadda R. Vasudevan E. Celnic P. Bastian A. (2012). Modulating locomotor adaptation with cerebellar stimulation. J. Neurophysiol. 107, 2950–2957 10.1152/jn.00645.2011 22378177 PMC3378372

[B30] Jeffery D. T. Norton J. A. Roy F. D. Gorassini M. A. (2007). Effects of transcranial direct current stimulation on the excitability of the leg motor cortex. Exp. Brain Res. 182, 281–287 10.1007/s00221-007-1093-y 17717651

[B31] Kantak S. S. Sullivan K. J. Fisher B. E. Knowlton B. J. Winstein C. J. (2010). Neural substrates of motor memory consolidation depend on practice structure. Nat. Neurosci. 13, 923–925 10.1038/nn.2596 20622872

[B32] Kantak S. S. Winstein C. J. (2012). Learning-performance distinction and memory processes for motor skills: a focused review and perspective. Behav. Brain Res. 228, 219–231 10.1016/j.bbr.2011.11.028 22142953

[B33] Kim D.-Y. Lim J.-Y. Kang E. K. You D. S. Oh M.-K. Oh B.-M. Paik N.-J. (2010). Effect of transcranial direct current stimulation on motor recovery in patients with subacute stroke. Am. J. Phys. Med. Rehabil. 89, 879–886 10.1097/PHM.0b013e3181e7201a 20962598

[B34] Kleim J. A. (2011). Neural plasticity and neurorehabilitation: teaching the new brain old tricks. J. Commun. Disord. 44, 521–528 10.1016/j.jcomdis.2011.04.006 21600589

[B35] Kleim J. A. Jones T. A. (2008). Principles of experience-dependent neural plasticity: implications for rehabilitation after brain damage. J. Speech Lang. Hear. Res. 51, S225–S239 10.1044/1092-4388(2008/018) 18230848

[B36] Krakauer J. W. (2006). Motor learning: its relevance to stroke recovery and neurorehabilitation. Curr. Opin. Neurol. 19, 84–90 10.1097/01.wco.0000200544.29915.cc 16415682

[B37] Krakauer J. W. Carmichael S. T. Corbett D. Wittenberg G. F. (2012). Getting neurorehabilitation right: what can be learned from animal models? Neurorehabil. Neural Repair. [Epub ahead of print]. 22466792 10.1177/1545968312440745 PMC4554531

[B38] Kuo M.-F. Grosch J. Fregni F. Paulus W. Nitsche M. A. (2007). Focusing effect of acetylcholine on neuroplasticity in the human motor cortex. J. Neurosci. 27, 14442–14447 10.1523/JNEUROSCI.2391-07.2007 18160652 PMC6673455

[B39] Kuo M.-F. Paulus W. Nitsche M. A. (2008). Boosting focally-induced brain plasticity by dopamine. Cereb. Cortex 18, 648–651 10.1093/cercor/bhm098 17591596

[B40] Liebetanz D. Nitsche M. A. Tergau F. Paulus W. (2002). Pharmacological approach to the mechanisms of transcranial DC-stimulation-induced after-effects of human motor cortex excitability. Brain 125, 2238–2247 10.1093/brain/awf238 12244081

[B41] Lin K. C. Wu C. Y. Liu J. S. (2008). A randomized controlled trial of constraint-induced movement therapy after stroke. Acta Neurochir. Suppl. 101, 61–64 10.1007/978-3-211-78205-7_10 18642635

[B42] Lindenberg R. Renga V. Zhu L. L. Nair D. Schlaug G. (2010). Bihemispheric brain stimulation facilitates motor recovery in chronic stroke patients. Neurology 75, 2176–2184 10.1212/WNL.0b013e318202013a 21068427 PMC3013585

[B43] Lisman J. E. (2001). Three Ca2+ levels affect plasticity differently: the LTP zone, the LTD zone and no man’s land. J. Physiol. (Lond.) 532, 285–285 10.1111/j.1469-7793.2001.0285f.x 11306649 PMC2278561

[B44] Madhavan S. Rogers L. M. Stinear J. W. (2010). A paradox: after stroke, the non-lesioned lower limb motor cortex may be maladaptive. Eur. J. Neurosci. 32, 1032–1039 10.1111/j.1460-9568.2010.07364.x 20722719 PMC2943014

[B45] Madhavan S. Stinear J. W. (2010). Focal and bidirectional modulation of lower limb motor cortex using anodal transcranial direct current stimulation. Brain Stimul. 3, 42–50 10.1016/j.brs.2009.06.005 20161639 PMC2818023

[B46] Madhavan S. Weber K. A. II Stinear J. W. (2011). Non-invasive brain stimulation enhances fine motor control of the hemiparetic ankle: implications for rehabilitation. Exp. Brain Res. 209, 9–17 10.1007/s00221-010-2511-0 21170708

[B47] Maxwell J. P. Masters R. S. Eves F. F. (2003). The role of working memory in motor learning and performance. Conscious. Cogn. 12, 376–402 10.1016/S1053-8100(03)00005-9 12941284

[B48] Miranda P. C. Lomarev M. Hallett M. (2006). Modeling the current distribution during transcranial direct current stimulation. Clin. Neurophysiol. 117, 1623–1629 10.1016/j.clinph.2006.04.009 16762592

[B49] Muellbacher W. Richards C. Ziemann U. Wittenberg G. Weltz D. Boroojerdi B. Cohen L. Hallett M. (2002). Improving hand function in chronic stroke. Arch. Neurol. 59, 1278–1282 10.1001/archneur.59.8.1278 12164724

[B50] Nair D. G. Renga V. Lindenberg R. Zhu L. Schlaug G. (2011). Optimizing recovery potential through simultaneous occupational therapy and non-invasive brain-stimulation using tDCS. Restor. Neurol. Neurosci. 29, 411–420 22124031 10.3233/RNN-2011-0612 PMC4425274

[B51] Nitsche M. A. Fricke K. Henschke U. Schlitterlau A. Liebetanz D. Lang N. Henning S. Tergau F. Paulus W. (2003a). Pharmacological modulation of cortical excitability shifts induced by transcranial direct current stimulation in humans. J. Physiol. (Lond.) 553, 293–301 10.1113/jphysiol.2003.049916 12949224 PMC2343495

[B52] Nitsche M. A. Nitsche M. S. Klein C. C. Tergau F. Rothwell J. C. Paulus W. (2003b). Level of action of cathodal DC polarisation induced inhibition of the human motor cortex. Clin. Neurophysiol. 114, 600–604 10.1016/S1388-2457(03)00235-9 12686268

[B53] Nitsche M. A. Schauenburg A. Lang N. Liebetanz D. Exner C. Paulus W. Tergau F. (2003c). Facilitation of implicit motor learning by weak transcranial direct current stimulation of the primary motor cortex in the human. J. Cogn. Neurosci. 15, 619–626 10.1162/089892903321662994 12803972

[B54] Nitsche M. A. Kuo M.-F. Grosch J. Bergner C. Monte-Silva K. Paulus W. (2009a). D(1)-Receptor impact on neuroplasticity in humans. J. Neurosci. 29, 2648–2653 10.1523/JNEUROSCI.5366-08.2009 19244540 PMC6666237

[B55] Nitsche M. A. Kuo M.-F. Karrasch R. Waechter B. Liebetanz D. Paulus W. (2009b). Serotonin affects transcranial direct current-induced neuroplasticity in humans. Biol. Psychiatry 66, 503–508 10.1016/j.biopsych.2009.03.022 19427633

[B56] Nitsche M. A. Paulus W. (2000). Excitability changes induced in the human motor cortex by weak transcranial direct current stimulation. J. Physiol. (Lond.) 527(Pt 3), 633–639 10.1111/j.1469-7793.2000.t01-1-00633.x 10990547 PMC2270099

[B57] Nitsche M. A. Paulus W. (2001). Sustained excitability elevations induced by transcranial DC motor cortex stimulation in humans. Neurology 57, 1899–1901 10.1212/WNL.57.10.1899 11723286

[B58] Nitsche M. A. Seeber A. Frommann K. Klein C. C. Rochford C. Nitsche M. S. Fricke K. Liebetanz D. Lang N. Antal A. Paulus W. Tergau F. (2005). Modulating parameters of excitability during and after transcranial direct current stimulation of the human motor cortex. J. Physiol. (Lond.) 568, 291–303 10.1113/jphysiol.2005.092429 16002441 PMC1474757

[B59] Parazzini M. Fiocchi S. Rossi E. Paglialonga A. Ravazzani P. (2011). Transcranial direct current stimulation: estimation of the electric field and of the current density in an anatomical human head model. IEEE Trans. Biomed. Eng. 58, 1773–1780 10.1109/TBME.2011.2116019 21335303

[B60] Poreisz C. Boros K. Antal A. Paulus W. Poreisz C. Boros K. Antal A. Paulus W. (2007). Safety aspects of transcranial direct current stimulation concerning healthy subjects and patients. Brain Res. Bull. 72, 208–214 10.1016/j.brainresbull.2007.01.004 17452283

[B61] Priori A. Berardelli A. Rona S. Accornero N. Manfredi M. (1998). Polarization of the human motor cortex through the scalp. Neuroreport 9, 2257–2260 10.1097/00001756-199807130-00020 9694210

[B62] Purpura D. P. McMurtry J. G. (1965). Intracellular activities and evoked potential changes during polarization of motor cortex. J. Neurophysiol. 28, 166–185 14244793 10.1152/jn.1965.28.1.166

[B63] Reis J. Schambra H. M. Cohen L. G. Buch E. R. Fritsch B. Zarahn E. Celnik P. A. Krakauer J. W. (2009). Noninvasive cortical stimulation enhances motor skill acquisition over multiple days through an effect on consolidation. Proc. Natl. Acad. Sci. U.S.A. 106, 1590–1595 10.1073/pnas.0805413106 19164589 PMC2635787

[B64] Rioult-Pedotti M. S. Friedman D. Donoghue J. P. (2000). Learning-induced LTP in neocortex. Science 290, 533–536 10.1126/science.290.5491.533 11039938

[B65] Rosen S. C. Stamm J. S. (1972). Transcortical polarization – facilitation of delayed response performance by monkeys. Exp. Neurol. 35, 282–286 10.1016/0014-4886(72)90154-9 4624141

[B66] Rosenkranz K. Nitsche M. A. Tergau F. Paulus W. (2000). Diminution of training-induced transient motor cortex plasticity by weak transcranial direct current stimulation in the human. Neurosci. Lett. 296, 61–63 10.1016/S0304-3940(00)01621-9 11099834

[B67] Rossi C. Sallustio F. Di Legge S. Stanzione P. Koch G. (2012). Transcranial direct current stimulation of the affected hemisphere does not accelerate recovery of acute stroke patients. Eur. J. Neurol. [Epub ahead of print]. 10.1111/j.1468-1331.2012.03703.x 22448901

[B68] Rossini P. M. Calautti C. Pauri F. Baron J. C. (2003). Post-stroke plastic reorganisation in the adult brain. Lancet Neurol. 2, 493–502 10.1016/S1474-4422(03)00485-X 12878437

[B69] Sadleir R. J. Vannorsdall T. D. Schretlen D. J. Gordon B. (2010). Transcranial direct current stimulation (tDCS) in a realistic head model. Neuroimage 51, 1310–1318 10.1016/j.neuroimage.2010.03.052 20350607

[B70] Seidler R. D. (2010). Neural correlates of motor learning, transfer of learning, and learning to learn. Exerc. Sport Sci. Rev. 38, 3–9 10.1097/JES.0b013e3181c5cce7 20016293 PMC2796204

[B71] Serrien D. J. Strens L. H. Cassidy M. J. Thompson A. J. Brown P. (2004). Functional significance of the ipsilateral hemisphere during movement of the affected hand after stroke. Exp. Neurol. 190, 425–432 10.1016/j.expneurol.2004.08.004 15530881

[B72] Shmuelof L. Krakauer J. W. (2011). Are we ready for a natural history of motor learning? Neuron 72, 469–476 10.1016/j.neuron.2011.10.017 22078506 PMC3389513

[B73] Siebner H. R. Lang N. Rizzo V. Nitsche M. A. Paulus W. Lemon R. N. Rothwell J. C. Siebner H. R. Lang N. Rizzo V. Nitsche M. A. Paulus W. Lemon R. N. Rothwell J. C. (2004). Preconditioning of low-frequency repetitive transcranial magnetic stimulation with transcranial direct current stimulation: evidence for homeostatic plasticity in the human motor cortex. J. Neurosci. 24, 3379–3385 10.1523/JNEUROSCI.5316-03.2004 15056717 PMC6730024

[B74] Stagg C. J. Best J. G. Stephenson M. C. O’Shea J. Wylezinska M. Kincses Z. T. Morris P. G. Matthews P. M. Johansen-Berg H. (2009). Polarity-sensitive modulation of cortical neurotransmitters by transcranial stimulation. J. Neurosci. 29, 5202–5206 10.1523/JNEUROSCI.4432-08.2009 19386916 PMC6665468

[B75] Stagg C. J. Jayaram G. Pastor D. Kincses Z. T. Matthews P. M. Johansen-Berg H. (2011). Polarity and timing-dependent effects of transcranial direct current stimulation in explicit motor learning. Neuropsychologia 49, 800–804 10.1016/j.neuropsychologia.2011.02.009 21335013 PMC3083512

[B76] Tanaka S. Hanakawa T. Honda M. Watanabe K. (2009). Enhancement of pinch force in the lower leg by anodal transcranial direct current stimulation. Exp. Brain Res. 196, 459–465 10.1007/s00221-009-1863-9 19479243 PMC2700246

[B77] Tanaka S. Takeda K. Otaka Y. Kita K. Osu R. Honda M. Sadato N. Hanakawa T. Watanabe K. (2011). Single session of transcranial direct current stimulation transiently increases knee extensor force in patients with hemiparetic stroke. Neurorehabil. Neural Repair 25, 565–569 10.1177/1545968311402091 21436391

[B78] Traversa R. Cicinelli P. Pasqualetti P. Filippi M. Rossini P. M. (1998). Follow-up on interhemispheric differences of motor evoked potentials from the “affected” and “unaffected” hemispheres in human stroke. Brain Res. 803, 1–8 10.1016/S0006-8993(98)00505-8 9729235

[B79] Ungerleider L. G. Doyon J. Karni A. (2002). Imaging brain plasticity during motor skill learning. Neurobiol. Learn. Mem. 78, 553–564 10.1006/nlme.2002.4091 12559834

[B80] Ward N. S. Brown M. M. Thompson A. J. Frackowiak R. S. (2003). Neural correlates of outcome after stroke: a cross-sectional fMRI study. Brain 126, 1430–1448 10.1093/brain/awg145 12764063 PMC3717456

[B81] Willingham D. B. (1998). A neuropsychological theory of motor skill learning. Psychol. Rev. 105, 558–584 10.1037/0033-295X.105.3.558 9697430

[B82] Yen C.-L. Wang R.-Y. Liao K.-K. Huang C.-C. Yang Y.-R. (2008). Gait training-induced change in corticomotor excitability in patients with chronic stroke. Neurorehabil. Neural Repair 22, 22–30 17507641 10.1177/1545968307301875

[B83] Zaehle T. Sandmann P. Thorne J. D. Jaencke L. Herrmann C. S. (2011). Transcranial direct current stimulation of the prefrontal cortex modulates working memory performance: combined behavioural and electrophysiological evidence. BMC Neurosci. 12, 2 10.1186/1471-2202-12-S1-O2 21211016 PMC3024225

[B84] Zimerman M. Heise K. F. Hoppe J. Cohen L. G. Gerloff C. Hummel F. C. (2012). Modulation of training by single-session transcranial direct current stimulation to the intact motor cortex enhances motor skill acquisition of the paretic hand. Stroke. [Epub ahead of print]. 22618381 10.1161/STROKEAHA.111.645382 PMC4879963

